# Low and high frequency tonal threshold audiometry: comparing hearing thresholds between smokers and non-smokers

**DOI:** 10.1016/S1808-8694(15)30527-9

**Published:** 2015-10-18

**Authors:** Daniela Cecílio Capra Marques de Oliveira, Marco Antonio de Melo Tavares de Lima

**Affiliations:** 1 MSc student - Graduate Studies in Medicine - General Surgery - Otorhinolaryngology - Federal University of Rio de Janeiro - UFRJ, ENT physician; 2 PhD in Medicine - UNIFESP. Associate Professor of Otorhinolaryngology and Ophthalmology - Medical School of the Federal University of Rio de Janeiro - UFRJ

**Keywords:** audiometry, auditory threshold, hearing loss, smoking, high-frequency

## Abstract

Cigarette smoking can cause many potentially fatal diseases and worsen others. Numerous studies have shown the relationship between smoking and hearing loss. However, the increase in auditory threshold in high frequency arising from smoking has been very little described.

**Aim:**

to compare low and high frequency auditory thresholds among a group of smoking and non-smoking male individuals between 18 and 40 years.

**Study design:**

Cross-sectional.

**Materials and Methods:**

by means of low and high frequency tonal threshold audiometry we studied 30 male individuals between 18 and 40 years and 30 non-smokers of matching age and gender.

**Results:**

auditory thresholds were different between smokers and non-smokers, being worse in the former. Although within normal ranges, auditory thresholds in low frequencies were higher among smokers. In high frequencies we noticed a marked increase in auditory thresholds among smokers.

**Conclusion:**

we found statistically significant difference in auditory thresholds in low and high frequencies, among young male individuals, smokers and non-smokers, being worse in the former.

## INTRODUCTION

Cigarette smoking is associated with numerous potentially fatal diseases, such as head and neck cancer, lung cancer, atherosclerosis, coronary and heart diseases, and others. Moreover, the problems caused by smoking are long known^1^. Although cigarette smoking has been on the decline in most developed countries, its global use has increased in 50% during the period of 1975 to 1996, especially because of the increase in cigarette smoking in developing countries. In Brazil, about 200 thousand people die annually - very likely because of the late effects of tobacco smoking^2^.

Approximately one hundred thousand young people start smoking every day^3^. Smoking is a widespread habit in this population and the damage caused by the inhaled substances has been increasingly studied^4^.

There is strong evidence that smoking can cause hearing loss. Numerous authors have reported on the damaging effects of the tobacco smoke on the cochlea and the increase in auditory thresholds in the low frequencies among smokers^5^, ^6^, ^7^.

The arterial coating is damaged by abnormal increases in the levels of blood carboxyhemoglobin among smokers. With a reduction in the blood supply of oxygen, first there are microlesions to the vessel walls - which favor the deposit of atheromatous plaques and the development of larger and elevated lesions. Arterial wall lesions reduce vessel diameter, thus reducing blood flow to the area it nourishes^8^. Moreover, nicotine can have a direct ototoxic effect and cause cochlear ischemia for increasing the carboxyhemoglobin production, favoring vasospasm, promoting atherosclerosis and increasing blood viscosity^9^.

Cochlear blood flow interruption and the consequent reduction in oxygen levels are the main pathophysiological mechanisms responsible for hearing loss in smokers^10^. Carbon monoxide may also act directly on cochlear metabolism and cause alterations to the action potentials generated by auditory nerve fibers. Another effect reported about the carbon monoxide on the inner ear was the metabolic exhaustion of the succinate dehydrogenase enzyme, implicated in the Krebs cycle of the inner ear cells, especially of the outer hair cells, and the oxidation of nervous structures for the production of free radicals^11^. Nonetheless, most authors study threshold increases only in frequencies up to 8 kHz. Smoking-related hearing loss in frequencies higher than 8 kHz is not much studied.

New equipment and test methods brought about other perspectives for the investigation of auditory damage arising from the action of many degenerative etiological agents^12^. Among them, we can stress High Frequency Tonal Audiometry (HFTA). Through it we can assess auditory sensitivity in a broader spectrum, in the frequencies above 8 kHz, allowing for new studies associated to the early diagnosis of auditory damage^13^, ^14^. The literature describes that HFTA can help in the early diagnosis of auditory damage stemming from aging, ototoxicity and high levels of sound pressure,^15^, ^16^ and the damage to the higher frequencies are perceived earlier on than those involving the lower frequencies^17^.

The goal of the present study is to compare the auditory threshold in low and high frequencies, within a group of male smokers and non-smokers, with ages between 18 and 40 years.

## MATERIALS AND METHODS

A cross-sectional study was carried out of the observational and exploratory descriptive type. The clinical research protocol was handed out to the Ethics in Research Committee (CEP) of the institution where the study was carried out, under register # 198/06, and was approved under approval # 912/06, dated on November 22, 2006, thus fulfilling the necessary requirements to perform research in human beings.

The equipment used to perform conventional and high frequency audiometry was the AMPLAID, model 460 audiometer. For conventional audiometry we used the Telephonics 296D 100-1 earphone, and for the high frequency audiometry, we used the HD-520 II phone.

From July of 2007 through January of 2008, we assessed 65 individuals. Five were taken off the study fore having at least one exclusion criteria. The remaining individuals were distributed in two distinct groups, 30 individuals in the non-smoking group (NSG) and 30 in the smoking group (SG).

In the Smoking Group (SG) we had male individuals with minimum age of 18 and maximum of 40 years, active smokers for at least five years. In the non-smoking group (NSG) there were male individuals, with minimum age of 18 and maximum age of 40 years who had never smoked.

We took off the study those individuals who had ear disorders, tinnitus and/or dizziness, hearing loss, prior ear surgery, otoscopic alterations, professional exposure to high sound pressure levels, conventional audiometry auditory thresholds higher than 25 dB SL in one or more frequencies, systemic arterial hypertension, diabetes mellitus and/or neurologic disease, illiterate individuals or those with visual disorders.

The volunteers who presented themselves for the study were met by the researcher, who explained the goal of the study, applied the semi-structured questionnaire and performed the otoscopy and the audiometric tests. All the volunteers read and signed the Informed Consent Form.

After the interview and otoscopy, the volunteer was referred to audiometric evaluation. Initially, the auditory thresholds were obtained by air conduction, in the frequencies of 0.25 kHz; 0.5 kHz; 1 kHz; 2 kHz; 4 kHz; 6 kHz; and 8 kHz, in both ears, always starting with the right ear. The 3 kHz frequency was not tested because the equipment is unable to evaluate it. For future analyses of this study, such set of frequencies started to be called low frequencies (LF).

In order to establish audiometric thresholds by air conduction we used the descending technique. At each tone detection response, the sound intensity was reduced in 10 dB until the individual no longer responded to the sound. From this intensity, the ascending technique was used and the sound intensity was higher in 5 dB intervals until the individual detected the response. The auditory threshold corresponds to the lowest sound intensity heard by the individual in each frequency. The individuals with auditory thresholds lower than or equal to 25 dB SL in the LF (ANSI, S. 3-6) were, afterwards, submitted to HFTA.

For this test we used the same equipment and sound booth used in conventional audiometry. Only the earphones were changed. The technique used to obtain the thresholds was also the same.

We obtained the auditory thresholds by air conduction in the frequencies of 9 kHz; 10 kHz; 11.2 kHz; 12.5 kHz; 14 kHz; 16 kHz; and 18 kHz. We chose these frequencies because they have international calibration standards which were used in the equipment configuration. For future analyses of this study, this set of frequencies started to be called high frequencies (HF).

These stages were realized at the same time and in the same way for SG and NSG individuals.

Later on, statistical descriptive and comparative studies were carried out with the groups. We used the ANOVA Variance Test with a double repetition factor which allows for the analysis of values and comparison between them, establishing or not the statistical significant differences.

## RESULTS

Initially, we calculated the mean and standard deviation of age among individuals belonging to the SG and NSG (Table 1).

**Table 1:** Mean and standard deviation (SD) values of the age variable between non-smokers (NSG) and smokers (SG).

	GNT	GT
Mean	27,53	30,53
SD	6,34	5,62
Minimum Age	18	21
Maximum Age	40	40
Count	30	30

The mean values of the auditory thresholds in LF and HF obtained from the RE were compared to the mean values from the LE, first in the NSG. We then employed the ANOVA variance test with a double repetition factor (significance level of 0.05), which checks whether or not a group of values have statistical significance among them, and the p-value was obtained equals; 0.0047, showing that there are statistically significant differences between the RE and LE mean values in the NSG individuals (Table 2).

**Table 2:** Comparison of the Right Ear (RE) and Left Ear (LE) auditory threshold mean values among non-smokers (NSG).

kHz	0,25	0,5	1	2	4	6	8	9	10	11.2	12.5	14	16	18
NSG RE														
Count	30	30	30	30	30	30	30	30	30	30	30	30	30	30
Mean	10,8	11,0	9,7	6,5	8,5	8,8	4,3	6,0	6,2	9,8	5,7	- 0,7	5,7	19,2
NSG LE														
Count	30	30	30	30	30	30	30	30	30	30	30	30	30	30
Mean	12,3	10,5	8,8	5,3	10,7	9,7	5,0	13,5	10,2	11,0	7,3	4,7	8,7	23,0
TOTAL														
Count	60	60	60	60	60	60	60	60	60	60	60	60	60	60
Mean	11,6	10,8	9,3	5,9	9,6	9,3	4,7	9,8	8,2	10,4	6,5	2,0	7,2	21,1
ANOVA														
FVa	SQ	gl	Q	F	P value	Critical F								
Sample	911,5	1,0	911,5	8,0	0,0047b	3,9								

Variation Source

p-value < 0.05= threshold mean values (in LF and HF), between RE and LF, in the NSG are statistically different.

Following that, we compared the mean values of the thresholds obtained in LF and HF, between the RE and LE in the SG. We employed again the ANOVA - variance test with double repetition factor (significance level of 0.05) among threshold means, and we found a p value of 0.052 showing that there is no statistical difference (p>0.05) among the mean values of the RE and LE thresholds in the SG individuals (Table 3).

**Table 3:** Comparison of the Right Ear (RE) and Left Ear (LE) auditory threshold mean values among smokers (SG).

kHz	0,25	0,5	1	2	4	6	8	9	10	11.2	12.5	14	16	18
SG RE														
Count	30	30	30	30	30	30	30	30	30	30	30	30	30	30
Mean	14,67	15,67	10,67	9,83	12,17	11,67	9,50	14,67	18,83	23,50	21,17	17,00	26,83	34,17
SG LE														
Count	30	30	30	30	30	30	30	30	30	30	30	30	30	30
Mean	12,8	14,0	10,5	7,8	9,7	7,7	8,7	13,8	15,7	21,7	20,7	15,7	24,7	31,8
TOTAL														
Count	60	60	60	60	60	60	60	60	60	60	60	60	60	60
Mean	13,8	14,8	10,6	8,8	10,9	9,7	9,1	14,3	17,3	22,6	20,9	16,3	25,8	33,0
ANOVA														
FVa	SQ	gl	MQ	F	p-value	Critical F								
Sample	678,6	1	678,6	3,8	0,052b	3,85								

Variation Source

p-value > 0.05: the mean threshold values from smokers, between the RE and the LE, are statistically similar.

Because of the statistically significant difference found among the thresholds of the RE and LE in the NSG, we chose to compare the auditory thresholds between the SG and NSG, separately for each ear.

Thus, we compared the mean thresholds of the RE between the SG and the NSG and, later on, the mean auditory thresholds of the LE between the SG and the NSG.

The RE auditory threshold values in the SG and NSG individuals were compared by employing the ANOVA - variance test, with a double repetition factor (significance level of 0.05). We then obtained a p value equals; 0.0000 showing a statistically significant difference in LF and HF in the mean threshold values of the RE between the SG and the NSG (Table 4).

**Table 4:** Comparison of the right ear (RE) auditory thresholds among non-smokers (NSG) and smokers (SG).

kHz	0,25	0,5	1	2	4	6	8	9	10	11,2	12,5	14	16	18
SG RE														
Count	30	30	30	30	30	30	30	30	30	30	30	30	30	30
Mean	14,3	15,7	10,3	9,5	11,5	11,0	9,2	14,0	17,8	22,7	20,0	15,8	26,5	33,3
NSG RE														
Count	30	30	30	30	30	30	30	30	30	30	30	30	30	30
Mean	10,8	11,0	9,67	6,50	8,50	8,83	4,3	6,00	6,17	9,83	5,67	-0,67	5,67	19,1
Total														
Count	60	60	60	60	60	60	60	60	60	60	60	60	60	60
Mean	12,5	13,3	10,0	8,0	10,0	9,9	6,7	10,0	12,0	16,2	12,8	7,5	16,0	26,2
ANOVA														
FV a	SQ	gl	MQ	F	p-value	Critical F								
Sample	15471,5	1	15471,5	103,0	0,000b	3,85								

Source of variation

p-value < 0.05: there is a statistically significant difference among the RE and LE threshold mean values between the NSG and the SG.

**Graph 1:**
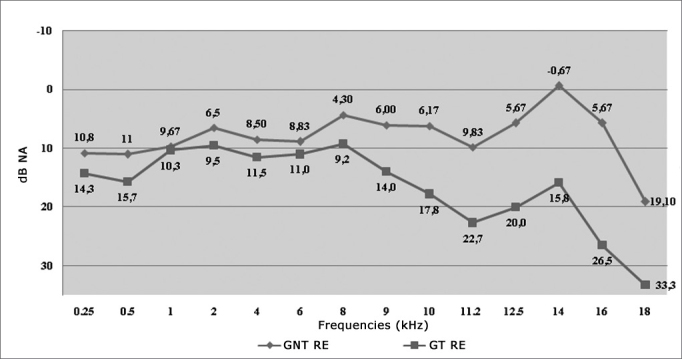
Variation of the Right Ear (RE) auditory threshold mean values among non-smokers (NS) and smokers (SG).

Graph 1 shows the variation of the RE threshold mean values between the groups studied. We observed higher auditory thresholds in the individuals from the SG and a more pronounced difference in the HF.

In the LE, the auditory thresholds between the groups were also compared using the ANOVA - variance test with a double repetition factor (0.05 significance level) and p value equals; 0.0000, also showing a statistically significant difference among the threshold mean values in LF and HF in the LE, between smokers and non-smokers (Table 5).

**Table 5:** Comparison of the left ear (LE) auditory thresholds among non-smokers (NSG) and smokers (SG).

kHz	0,25	0,5	1	2	4	6	8	9	10	11,2	12,5	14	16	18
SG LE														
Count	30	30	30	30	30	30	30	30	30	30	30	30	30	30
Mean	12,83	14,0	10,50	7,83	9,67	7,67	8,67	13,8	15,6	21,6	20,6	15,6	24,6	31,8
NSG LE														
Count	30	30	30	30	30	30	30	30	30	30	30	30	30	30
Mean	12,3	10,5	8,8	5,3	10,7	9,7	5,0	13,5	10,2	11,0	7,3	4,7	8,7	23,0
Total														
Count	60	60	60	60	60	60	60	60	60	60	60	60	60	60
Sum	755	735	580	395	610	520	410	820	775	980	840	610	1000	1645
Mean	12,6	12,3	9,7	6,6	10,2	8,7	6,8	13,7	12,9	16,3	14,0	10,2	16,7	27,4
ANOVA														
FVa	SQ	gl	MQ	F	p-value	Critical F								
Sample	5946,6	1	5946,7	41,78	0,000b	3,85								

**Graph 2:**
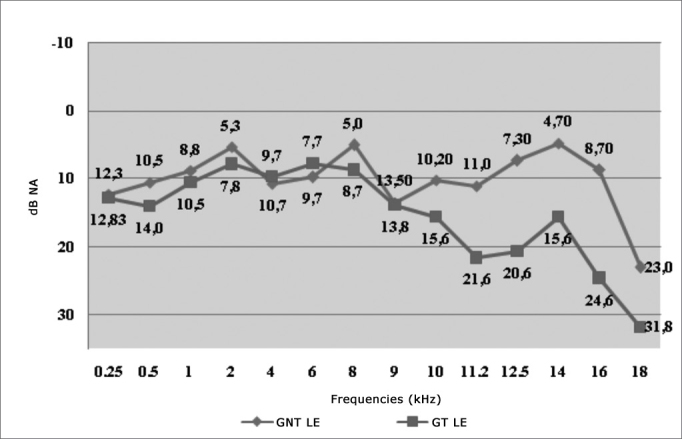
Variation of the Left Ear (LE) auditory threshold mean values among non-smokers (NS) and smokers (SG).

Graph 2 depicts the variation curve of the auditory threshold mean values for the LE for LF and HF, among smokers and non-smokers. We can observe that the SG thresholds are higher than those in the NSG. Moreover, the difference between the auditory threshold mean values is higher in the HF.

Results show that the mean auditory threshold values in the RE were different between SG and NSG. In the same way, the mean values of the LE thresholds also present statistically significant difference between the groups.

## DISCUSSON

In the present investigation, the mean age of individuals in the NSG was 27.53 years and the SG mean age was 30.53. In the literature we find the most varied age ranges being studied alone or together, and such fact makes it difficult to make an isolate analysis of the effects of the substances contained in the cigarette smoke on the auditory organs. In this case, age can be considered a confounding factor among the results obtained in these papers^6^, ^7^, ^8^, ^18^, since it has been well described that aging causes an increase in auditory thresholds because of the degeneration of cochlear sensorial organs^19^, ^20^, ^21^, and the stria vascularis atrophy is responsible for the most characteristic changes associated with aging on the human cochlea^22^. In order to rule out the influence of this factor, we chose to form groups of individuals with up to 40 years of age.

Moreover, it is known that hormonal variations can influence the values of auditory thresholds in women^23^. Aiming at neutralizing the interference of this other factor and obtaining the most comparable possible values, we made up groups of men only.

From there, we analyzed and compared the auditory thresholds between the RE and the LE separately in each group.

We obtained a statistically significant difference between RE and LE thresholds in the NSG, especially in the frequencies of 4, 9, 10 and 14 kHz. Contrary to this, this difference was not significant in the SG. SÁ et al. also found statistically significant differences between the RE and LE; however, only in the 11 kHz and 12 kHz frequencies, among male non-smoker youngsters, and such difference they deem having occurred randomly^24^. On the other hand, other authors described the lack of significant interaural difference in the auditory thresholds between the RE and LE obtained in their studies^8^, ^14^. We have also found authors who reported that the mean values obtained for the RE and LE together, without analyzing a possible significant interaural difference^15^, ^19^, ^27^. This difference in the auditory thresholds between the RE and LE in the NSG was an intriguing fact for which we have not found any explanation so far in the literature studied. Future studies may clear up this finding.

As already mentioned, the difference in thresholds between the RE and the LE was not significant in the SG. Reviewing the literature, we did not find any paper mentioning the separate analysis of the auditory thresholds between the RE and LE among smokers.

Because of the statistical difference found among the threshold mean values from the RE and LE in the NSG, we compared the auditory thresholds between the NSG and the SG separately for each ear.

We found a statistically significant difference in LF and HF, in the RE auditory thresholds between the SG and the NSG. By the same token, we found a statistically significant difference in the auditory thresholds for the RE between the NSG and the SG, for LF and HF. For both ears, the SG auditory thresholds were higher.

The raise in auditory thresholds in LF among smokers is abundantly reported in the literature. Numerous authors also found associations between smoking and hearing loss in LF^6^, ^9^, ^10^, ^22^, 25, 26, 27, 28, 29. Hearing loss in HF can be 1 to 1.33 times higher among smokers^24^.

In relation to the AF studies among smokers, after an extensive literature review, we found only one paper reporting the relation between the effects of smoking on the auditory thresholds in HF. In the present investigation, the authors did not find statistically significant differences among the auditory threshold mean values in HF between smokers and non-smokers. Although the inclusion criteria used by these authors were very similar to the ones used in the present study, a piece of data we find very relevant, may explain the reason for the absence of significant differences in the auditory thresholds between smokers and non-smokers in that study. The authors studied only those individuals who smoked the equivalent of one pack per day during six years, and such limit was not established in the present investigation. The literature describes the risk of hearing loss associated to the number of cigarettes smoked per day and the duration of cigarette exposure^28^. Moreover, the lower number of individuals in the groups can also have influenced that result and must be taken into account.

## CONCLUSION

In the present study we found a statistically significant difference in the low and high frequency auditory thresholds among young male non-smokers and smokers, being worse among the latter.
